# Pathway Regulation of p63, a Director of Epithelial Cell Fate

**DOI:** 10.3389/fendo.2015.00051

**Published:** 2015-04-28

**Authors:** Kathryn Yoh, Ron Prywes

**Affiliations:** ^1^Department of Biological Sciences, Columbia University, New York, NY, USA

**Keywords:** p63, epithelial cells, notch signalling, Wnt proteins, Hedgehog pathways, EGFR, epithelial–mesenchymal transition

## Abstract

The p53-related gene p63 is required for epithelial cell establishment and its expression is often altered in tumor cells. Great strides have been made in understanding the pathways and mechanisms that regulate p63 levels, such as the Wnt, Hedgehog, Notch, and EGFR pathways. We discuss here the multiple signaling pathways that control p63 expression as well as transcription factors and post-transcriptional mechanisms that regulate p63 levels. While a unified picture has not emerged, it is clear that the fine-tuning of p63 has evolved to carefully control epithelial cell differentiation and fate.

## Introduction

At first glance, the tumor suppressor p53 and its family member p63 seem quite similar in function and exhibit a high degree of evolutionary conservation. In particular, the DNA-binding domains are about 60% identical at the amino acid level; however, the adjacent domains and C-termini diverge drastically (1). While it was first thought that p63 and p53 could regulate similar sets of genes, it has become clear that these potent transcription factors possess some partially redundant functions, and some that are entirely unique (2–4).

p63 is also unlike its family member p53 in that it is rarely mutated in human cancers. Instead, mutations in p63 lead to disorders with ectodermal dysplasia such as ankyloblepharon-ectodermal dysplasia-clefting (AEC)/Hay–Wells syndrome, which can include symptoms like cleft lip/palate and skin erosions (5, 6). Other p63 syndromes can include split hand/foot malformation and alopecia, but cancer predisposition is generally not seen (7–9).

Due to differential promoter usage and splicing, there are at least six common isoforms. There are two classes that arise from different promoters, one with the N-terminal transactivation domain (TA), and the other set lacking the N-terminal transactivation domain (ΔN). While the ΔN form can be dominant negative to the TA isoforms (2), the ΔNp63α isoform has been shown to contain an alternate transcriptional activation domain, suggesting it can also directly activate target genes (3, 10). Alternative splicing of the 3′ end of the TA and ΔNp63 mRNAs produces the α, β, and γ isoforms, although only the α isoforms contain the sterile-α motif (SAM) domain and the transcription-inhibitory (TI) domain. Mutations in these domains can disrupt binding to the target Apobec-1-binding protein-1 (ABBP1), and deletion of both domains led to increased p21^Waf1/Cip1^ signaling, indicating that these domains can modulate target gene specificity (11, 12).

As to the specific functions of these isoforms, mouse models have been instrumental in providing us with clues. Two groups reported that p63^−/−^ mice were found to have severe limb and epithelial defects, including partial or missing epithelial stratification, and truncated forelimbs (13, 14). More recently, both the whole animal- and epidermal-specific deletion of ΔNp63α in mice led to skin erosions and impaired terminal differentiation of keratinocytes, demonstrating the importance of this isoform in the epithelial stratification process (15–17). It is possible that deregulation of p63 targets linked to cell–matrix adhesion and epithelial morphogenesis causes these skin abnormalities (18–20).

Furthermore, loss of epithelial cells in ΔNp63-null mice suggested that this isoform is essential for the establishment of epidermal progenitor cells (13). Pellegrini et al. (21) suggested that p63 is found in the stem cells of the proliferative compartment, but not in the transit amplifying keratinocytes that have exited the compartment. When it comes to the caudal endoderm, Pignon et al. (22) revealed that the p63-expressing cells are capable of differentiating into prostate, bladder, and colorectal epithelia. Another report found p63 to be essential for the proliferative ability and differentiation of the epidermis; however, in a thymic model, p63 was only required for clonogenicity but not for lineage commitment or differentiation (23, 24). Intriguingly, depletion of ΔNp63 or its target DGCR8, an miRNA processing factor, allowed keratinocytes to enter a multipotent stem cell state, suggesting that ΔNp63 is needed to maintain the keratinocyte differentiation state (25). Finally, an AEC-like mutation in p63 led to reduced proliferative and clonogenic potential in epithelial cells (26). Together these studies make a compelling case for p63 in the maintenance and regulation of epithelial stem cells.

Meanwhile, TAp63 ablation demonstrated that this isoform monitors the integrity of the germline after cellular stresses (27, 28). In particular, γ-irradiation was shown to induce tetramerization of TAp63α from inactive dimers, leading to greatly increased target binding ability (29), and inducing cell cycle arrest or an apoptotic response.

Yet, p63 levels are sometimes altered in tumors. Many groups have reported increased expression in cancers, especially in head and neck squamous cell carcinomas (HNSCC) (30, 31). Indeed, amplification or overexpression of p63 has frequently been observed in lung cancers, and more rarely in HNSCC (32–34). However, p63 expression is lost in more invasive prostate and breast cancers, and this loss is associated with worse prognosis in some cases (35, 36). It has been theorized that the tissue context, as well as the balance between TA and ΔN isoforms, could partially explain this dichotomy.

So how does p63 impact cancer formation? The last decade has seen a preponderance of direct targets unearthed, including adhesion-related β4 integrin, the tissue integrity factor Perp, the Notch ligands Jagged1 and Jagged2, keratins 5 and 14, and EGF receptor (18, 19, 37–41). Cancer-related targets like N-cadherin, Id3, MMP13, and Wnt-4 can be activated by p63; however, p63 can also induce Sharp1 and Cyclin G2 expression, which have been shown to be suppressors of breast cancer metastasis (42–45). Additionally, phosphorylated ΔNp63α was found to associate with components of the splicing machinery, as well as transcription factors SREBP1 and E2F1, in regulation of metabolic and cell cycle-related processes (46).

p63 is also known to regulate a diverse set of microRNAs. A prominent target is miR-205, a repressor of epithelial–mesenchymal transition (EMT) and metastasis in bladder and pancreatic cancers (36, 47, 48). In contrast to the role of miR-205, members of the miR-17 family (miR-17, miR-20b, and miR-106a) are regulated by p63 and Myc, and were found to target Rb, p21, and JNK2, suggesting that they are oncomirs (49–51). Additionally, p63 can repress the prominent cell cycle regulators miR-34a and miR-34c, thereby affecting cellular progression in a p53-independent manner (52).

A data mining approach also identified p63 and the p53-related p73 gene as key regulators of microRNAs differentially expressed in ovarian carcinomas, including miR-200a, miR-200b, and miR-429 (53). Similarly, mir-193a was repressed by both p63 and p73, although its induction leads to p73 inhibition (54). For more on p63 regulation of microRNAs, see the review by Candi et al. (55).

Taken together, p63, like p53 and p73, can regulate a host of processes, some of which are known regulators for or against tumor growth. As suggested by the opposite expression of p63 in different tumor types, the context of the cell type appears to be critical to which p63 targets have the dominant effects in each cell. Whether targets are differentially expressed or have different activities in different cell types needs to be investigated further.

**Figure 1:**
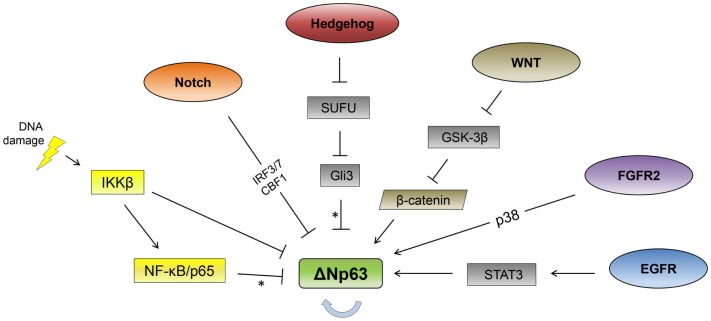
**Signaling pathway regulation of ΔNp63**. Signaling pathways reported to regulate ΔNp63 levels are indicated. The thick, blue arrow indicates autoregulation. *Note that the Hedgehog and NF-κB pathways repress ΔNp63 levels while simultaneously activating expression from the TAp63 promoter. See text for feedback regulation where p63 regulates multiple components of these pathways (not shown here).

As ΔNp63 is required for the formation of stratified epithelial layers and is the primary isoform expressed in the basal layer of epithelial tissues, it is subject to multiple modes of tissue-specific regulation (13, 14). As described below, a number of signaling pathways and transcription factors have been identified that affect p63 expression in epithelial cells (Figure 1).

## Notch Signaling

One prominent pathway is Notch, which can control epidermal differentiation as well as other developmental pathways (56, 57). Notch activation was found to suppress p63 expression in keratinocytes, ectodermal progenitor cells, and mammary epithelial cells (58–60). The repression in keratinocytes was dependent on the IRF3 and IRF7 transcription factors (59). In mouse mammary epithelial cells, the Notch-mediated repression of p63 functions through the CBF1/RBP-Jk transcription factor (60). In addition to these cases, there has been a report of Notch *activation* of p63 in fibroblasts (61), suggesting differing cell-specific modes of regulation.

The Notch-to-p63 pathway is subject to feedback regulation by ΔNp63, as it can activate Notch pathway gene expression (58, 60, 62). This loop could delineate the boundary between basal and luminal mammary cells as well as allow for ectodermal specification during development (58, 60).

As with p63 mutations, alterations in interferon regulatory factor 6 (IRF6) are associated with craniofacial abnormalities like cleft lip and/or palate (63, 64). Both IRF6 and p63 are required for normal palate development, so the finding that ΔNp63 induces IRF6 expression is logical, but surprisingly, IRF6 in turn causes proteasomal degradation of ΔNp63 (65, 66). Notch has also been found to activate IRF6 expression in keratinocytes (67). Together, these results suggest a Notch/p63/IRF6 axis regulates genes involved in epithelial development. Importantly, Notch, p63, and IRF6 genes were found mutated in about 30% of HNSCC cases, suggesting that this developmental pathway can be hijacked to promote tumor growth (68).

## Hedgehog Signaling

Hedgehog is another essential pathway for development (69, 70), and it is reported to regulate p63 expression. Hedgehog activation is seen in various cancers including lung, prostate, and breast (71, 72). Hedgehog ligands including Indian Hedgehog (IHH) can lead to activation of the Gli3 transcription factor, while absence of these ligands leads to a repressive form of the Gli3 transcription factor, termed Gli3^R^ (73, 74). This balance of Gli3 forms can control p63 isoform formation, as IHH induction of Gli3 actually upregulates TAp63 expression while reducing ΔNp63 promoter usage (75). Again, there is a regulatory loop here since TAp63 expression can increase IHH expression. Similarly, both TA and ΔNp63 β and γ isoforms can activate Sonic Hedgehog (SHH) expression and recently ΔNp63 was found to induce expression of Gli2 and the Hedgehog receptor Ptch1, affecting mammary stem cell renewal (76, 77). In addition, it was posited that some of the developmental defects observed in the p63^−/−^ mice may occur due to subsequent repression of SHH and other Hedgehog pathway genes (76). Other connections between the p63 and Hedgehog pathways include ΔNp63 activation of Gli2 and Gli3 as well as suppressor of fused (SUFU) (78–80). As SUFU is an inhibitor of the Gli proteins, these contrasting effects show the complexity of this signaling system. Nevertheless, together these results suggest a strong connection between the Hedgehog and p63 signaling pathways that could control normal epithelial differentiation or cancer progression.

## Wnt Signaling

Strikingly, mutations in the WNT genes also cause similar craniofacial abnormalities as p63 and IRF6 mutations (81, 82). Moreover, mutations in the Pbx genes in mice resulted in a similar phenotype and perturbed Wnt signaling (82). Further analysis demonstrated a Pbx-Wnt9b/Wnt3-p63-IRF6 signaling axis controlling development of the midfacial ectoderm (82). Chromatin immunoprecipitation and reporter genes suggested that p63 is directly regulated by the Wnt pathway through binding of Lef1/Tcf with β-catenin to a region between the TA and ΔNp63 promoters (82), although another report identified a β-catenin responsive site within the proximal ΔNp63 promoter (83). Recently, the Hedgehog pathway was also shown to be connected to craniofacial defects (84). Compound mutations in the Hedgehog pathway genes Hedgehog acyltransferase (Hhat) and Patched 1 (Ptch1) led to a cleft lip-like phenotype and these acted through reduced Wnt-p63-IRF6 signaling.

Analysis of keratinocyte differentiation has led to a different characterization of the p63, Wnt, and Notch signaling pathways. Knockdown of p63 caused reduced Wnt and Notch signaling (50, 51), suggesting that they lie downstream of p63 in contrast to the models of craniofacial development. This could be reconciled as part of a feedback regulation pathway as described above for Notch and p63. Additionally, the activation of Wnt and Notch by p63 may be dependent upon the availability of other transcription factors. For instance, the depletion of p63 led to reduced Myc gene expression via lowered Wnt/β-catenin and Notch signaling, and this is consistent with the requirement of both p63 and Myc for keratinocyte proliferation (50, 51). p63 was also found to regulate the expression of Myc and β-catenin in esophageal squamous cell carcinomas, suggesting the general functioning of a p63/β-catenin/Myc pathway in tumorigenesis (85). Finally, ΔNp63 was shown to upregulate the Wnt receptor Fzd7, leading to enhanced mammary stem cell formation and clonogenic potential (86).

## FGFR2/EGFR Pathways

Mutations in the FRGR2 gene (also known as KGFR) can also lead to craniofacial disorders such as cleft lip and Crouzon’s syndrome (87, 88). The splice variant FGFR2-2b is an epithelial-specific receptor for ligands like FGF1 and FGF7 (KGF), and is required for embryogenesis and adult tissue homeostasis (89). FGFR signaling and ΔNp63 can influence each other, as ΔNp63 activates expression of FGFR2 in thymic epithelial cells (90) and KGF-induced ΔNp63 expression in limbal epithelial cells (91). KGF’s effects on ΔNp63 require p38 MAPK, suggesting a novel pathway for regulation of p63 (91). Furthermore, mutations in p63 that cause AEC syndrome led to impaired FGFR2 gene expression and increased splicing of the mesenchymal FGFR2-2c isoform (11, 26). Together, the combination of FGFR2 activation of ΔNp63 and ΔNp63 induction of specific isoforms of FGFR2 are likely to lead to increased proliferation of specific epithelial cell types. This could enhance proliferation of progenitor cells, but might block progression of specific epithelial cancers.

Interestingly, FGFR2 can induce expression of the epithelial-specific transcription factor Elf5, and deletion of Elf5 causes altered expression of ΔNp63 in the luminal compartment of mouse mammary tissue (92–94). This suggests a pathway for cell type-specific expression of ΔNp63 mediated by Elf5.

The tyrosine kinase receptor EGFR has also been found to induce ΔNp63 expression. In one case, this was through phosphatidylinositol-3-kinase (PI3K) signaling in keratinocytes (42), while in two types of carcinomas EGFR activation of ΔNp63 was found to be mediated by STAT3 (95, 96). STAT3 was also required for ΔNp63 expression in limbal keratinocytes (97). The inhibition of the STAT3 growth-stimulatory pathway allowed the concomitant differentiation of the limbal keratinocytes, further suggesting the importance of ΔNp63 regulation in these and likely other epithelial cells. The PI3K and STAT3 pathways may be connected through mTOR signaling, as Ma et al. (62) found that PI3K activation of mTOR led to mTOR-dependent activation of the STAT3-p63-Jagged pathway. This highlights the interconnectedness of these signaling pathways, and the role of STAT3 as a key regulator of p63. However, a clear mechanism for how STAT3 directly regulates p63 remains to be determined.

## Regulation of ΔNp63 during the Epithelial to Mesenchymal Transition

Epithelial cells can undergo an EMT during development and during carcinogenesis, progressing to a more invasive and metastatic phenotype. This differentiation is thought to allow the cancerous cells greater motility and increased metastatic potential [see reviews by Thiery (98) and Kang and Massagué (99)]. The expression of ΔNp63 is repressed during this transition (100, 101). Transcription factors that can induce EMT include Snail, Slug (also known as Snail2), and Zeb1, and all of these can repress ΔNp63 in epithelial cells (100, 102–104). This inhibition, however, may be due to a feedback loop, as ΔNp63 expression can inhibit EMT by activation of miR-205, which suppresses Zeb1 and Zeb2 expression (36, 48).

Other transcription factors involved in control of EMT are Ovol1 and Ovol2 (85, 105). These factors can repress Zeb1 expression; however, it was also found that ΔNp63 expression increased in Ovol1- and Ovol2-deficient cells, and that Ovol2 could bind to several sites within the ΔNp63 promoter (85). Ovol2 may be upstream of ΔNp63 in an EMT-inducing pathway; alternatively, there may be feedback of Ovol2 to ΔNp63 (as there is with Zeb1 and ΔNp63) with ΔNp63 being an activator of Ovol2. In general, it remains to be characterized how ΔNp63 is regulated during EMT in different epithelial cell types.

## Transcription Factor Control

While we have mentioned a number of transcription factors as regulators of ΔNp63, a clear picture has yet to emerge on which factors are critical direct regulators of ΔNp63 and through which sequence elements they act near the ΔNp63 gene.

It is possible that multiple pathways regulate p63 through the C/EBP family of transcription factors, as they have been repeatedly found to regulate p63. C/EBPδ was found to bind to multiple regions of the ΔNp63 gene in human keratinocytes (106, 107). Antonini et al. (108, 109) assayed all conserved regions throughout the p63 gene and identified two, termed as C38 and C40, in the second intron of the ΔNp63 gene that affect expression in mouse keratinocytes. The C40 region was needed for expression in keratinocytes, while C38 provided repression during calcium-dependent differentiation. They found that C/EBPα and β bound to the C38 and C40 regions, and that overexpression of these factors repressed reporter gene expression. In addition, siRNA depletion of C/EBPα and β slightly increased p63 mRNA levels in differentiating cells, suggesting that C/EBPα and β are direct repressors of p63 expression. Furthermore, these investigators found AP-2 to be an activator of the C40 region and the POU domain protein Pou3f1 to be a repressor (108, 109). In contrast to the repression by C/EBPα and β described above, another group described a C/EBP site within the proximal human ΔNp63 promoter, which was required for expression in A431 epidermal carcinoma cells (100). C/EBPα was also found to positively activate a site within the mouse ΔNp63 promoter in mouse keratinocytes (110). Finally, after chemical stress, the cytosolic NAD(P)H:quinone oxidoreductase 1 (NQO1) was found to bind to and inhibit C/EBPα, partially accounting for its inhibition of ΔNp63 expression (110, 111). These contrasting effects of C/EBP may reflect different family members, DNA-binding sites or cell types used, suggesting that further studies are needed to better understanding of the roles these factors play in regulating p63.

Other transcription factors have also been found to regulate the p63 gene. An OCT4 binding site within the TAp63 promoter activates its expression, suggesting its involvement in stem cell regulation (112). Another pluripotency factor, Sox2, bound to p63 protein and localized with it to common gene loci in chromatin immunoprecipitation experiments. This binding occurred in squamous cell carcinoma cells, but not in embryonic stem cells, suggesting that p63 may co-opt pluripotency factors for differentiated cell-specific expression (105).

## p63 Autoregulation and Interaction with p53

p63 positively activates its own expression through binding to the C38 and C40 intronic enhancers as well as to its own proximal promoter (108, 109, 113). Overexpression of the ΔNp63γ isoform increased expression of ΔNp63α in HeLa cells, and of a promoter reporter gene in keratinocytes (108, 113). Overexpression of ΔNp63 was also found to increase expression of endogenous ΔNp63 in a nasopharyngeal carcinoma cell line where activation was dependent upon the STAT3 transcription factor (95). Whether binding of p63 to its promoter is direct or through another transcription factor, the evidence consistently shows that it positively feeds back to augment its own expression.

Initially, p63 expression was found to be suppressed by stresses, such as UV irradiation, that stimulate p53 expression (114–116). Binding of p53 to the ΔNp63 proximal promoter was detected in a mammary epithelial cell line, suggesting direct regulation by p53 of ΔNp63 expression (116). Mutant p53 proteins could also bind to the p63 protein in tumor cell lines and inhibit its activity (117), while in carcinoma cells it was shown that mutant p53 together with SMADs could sequester p63, resulting in inhibition of p63 and increased metastatic potential (45). While these results suggest that wild-type and mutant p53 can repress p63 expression and function, more work is needed to demonstrate the significance of this effect in human cancers, and exactly how this could contribute to tumorigenesis.

## Post-Transcriptional Regulation

p63 levels are also regulated by miRNA, ubiquitin-dependent proteasomal degradation, and protein phosphorylation. Notably, miR-203 can repress p63 expression in supra-basal epithelial cells, contributing to definition of the border between progenitor and differentiated epithelial cells (118, 119). In addition, miR-203 expression was activated during luminal mammary epithelial differentiation and ectopic expression of miR-203 stimulated EMT (120). These results suggest that miR-203 is an essential part of the epithelial differentiation pathway.

Other miRNAs have also been found to regulate p63 expression. miR-92 targets ΔNp63α and β in the HaCaT keratinocyte cell line and in myeloid cells, respectively, and miR-302 suppressed p63 expression in germ cells (121, 122). The apotosis stimulating protein of p53 (ASPP) family of p53 coactivators has similarities with protein phosphatases (123). A related family member iASPP (also known as PPP1R13L) is an inhibitor of apoptosis and can also bind to p63 (124). The expression of iASPP in the basal layer of skin cells is strikingly similar to that of p63, and knockdown of iASPP promoted epithelial differentiation (125). However, rather than regulating p63 by protein–protein interaction, Chikh et al. (125) found that iASPP inhibits the expression of two miRNAs, miR-574-3p and miR-720, which inhibit p63 expression. There is an auto-regulatory loop as p63 is needed for expression of these miRNAs and binds to the promoter of the iASPP gene. These experiments point to a critical role of iASPP and repression of its target miRNAs in maintenance of p63 expression and the epithelial phenotype.

p63 protein stability is also regulated by the ubiquitin–proteasome system, adding another layer of regulation (126). One example is p53-induced RING-H2 (Pirh2), an E3 ubiquitin ligase, which can directly bind to p63 and cause its poly ubiquitination and degradation in keratinocytes (127, 128). Pirh2 was also a transcriptional target of ΔNp63, establishing an auto-regulatory loop, and was required for epithelial differentiation. Another E3 ubiquitin ligase, Ring1B, part of the polycomb repressive complex 1 (PRC1), was found to target p63 (129). Ring1b is overexpressed in breast and pancreatic cancer cells (129, 130), suggesting a possible mechanism for p63 suppression in these tumors.

While p53 is stabilized by DNA damaging agents, such as UV irradiation, ΔNp63 is degraded (115, 131, 132). Two mechanisms related to the NF-κB pathway have been found to mediate this degradation. In the first, IKKβ binds to ΔNp63 and phosphorylates it to induce ubiquitination and degradation (133). A second mechanism is direct binding of the p65 subunit of NF-κB to ΔNp63 in cisplatin-treated cells, leading to proteasomal degradation of ΔNp63 (134). The reduction of ΔNp63 augmented activation of p53 target genes and may contribute to cell death in UV-damaged cells. NF-κB repression of p63 may also have a role in epithelial cell differentiation, as overexpression of the NF-κB factor p65 in epithelial cells led to p63 downregulation and increased EMT (103). ΔNp63 also bound to target genes with p65, suggesting that these two factors coordinately regulate a gene program promoting cell survival (135).

NF-κB can also activate the TAp63 promoter, suggesting that a shift to the TAp63 form could also underlie the DNA damage response (136). Again, there is an auto-regulatory loop where TAp63 activates p65 expression as well as stabilizes p65 protein by direct binding (137, 138).

An alternative mechanism for ΔNp63 degradation as part of the DNA damage response is phosphorylation on threonine 397 by the protein kinase HIPK2 (139). HIPK2 has previously been identified as a DNA damage-induced kinase targeting p53 (140), such that it provides a mechanism to coordinate p63 levels with p53 and other aspects of the DNA damage response. For more regulators of p63 protein stability, see the review by Li and Xiao (126).

## Conclusion

p63 has been termed as a master regulator of epithelial cells, and it is often suppressed in order for these cells to differentiate (21, 141, 142). We now understand more about how p63 is regulated, uncovering a large array of signaling pathways (Figure 1) and feedback regulation that controls expression of components of the signaling pathway as well as p63. Besides the processes of differentiation and development, p63 is also regulated during the DNA damage response, suggesting that it can mediate the more immediate fate of cells. The regulation of ΔNp63 expression, the predominant form in epithelial cells, includes transcriptional and post-transcriptional components. The relative importance of each pathway is still unclear and their usage will likely vary in different cell types and developmental stages. While there are multiple reports of some pathways and mechanisms, common regulatory sequence element(s) for control of the p63 gene across systems have yet to be established.

It will also be important to understand how modulation of p63 levels affects cancer formation. The combination of heterozygous p63 and p53 genotypes in mice yielded conflicting results, giving either greater or reduced tumor burdens (143, 144). Additionally, while ΔNp63 is often highly expressed or amplified in squamous carcinomas, other tumors such as esophageal adenocarcinomas and hepatocellular carcinomas generally lack expression (145–147). Naturally, these cancers arise from diverse tissues, but it is confounding that p63 can have oncogenic effects in some cases and tumor suppressive ones in others. As EMT is part of metastatic progression of some carcinomas, it is interesting that repression of p63 was seen during this differentiation process; is this regulation critical for progression of tumor cells to a more aggressive state? Further, which of the pathways described here, if any, are altered in cancer cells and modulate p63 levels in a critical manner?

Other open questions concern p63 promoter usage and splicing – what factors determine the balance of usage of the TA and ΔN promoters, and what governs the presence of different 3′ splicing isoforms? How does the balance of these 3′ isoforms lead to differences in development or oncogenesis? Finally, can the signaling pathways that control p63 levels be controlled to provide a therapeutic benefit in specific cancers? We can hope that the following years will bring a greater understanding of this master regulator of epithelial biology.

## Conflict of Interest Statement

The authors declare that the research was conducted in the absence of any commercial or financial relationships that could be construed as a potential conflict of interest. The Guest Associate Editor Wen Zhou declares that, despite being affiliated to the same institution as authors Ron Prywes and Kathryn Yoh, the review process was handled objectively and no conflict of interest exists.
